# Working and hypertension: gaps in employment not associated with increased risk in 13 European countries, a retrospective cohort study

**DOI:** 10.1186/1471-2458-14-536

**Published:** 2014-05-30

**Authors:** Juliet Rumball-Smith, Arijit Nandi, Jay S Kaufman

**Affiliations:** 1 Institute of Health Policy, Management and Evaluation, University of Toronto, 155 College St, Suite 425, Toronto, ON M5T 3M6, Canada; 2 Institute for Health and Social Policy and Department of Epidemiology, Biostatistics and Occupational Health, McGill University, 1130 Pine Avenue West, Montreal, QC H3A 1A3, Canada; 3 Department of Epidemiology, Biostatistics and Occupational Health, McGill University, 1020 Pine Avenue West, Montreal, QC H3A 1A2, Canada

**Keywords:** Hypertension, Unemployment, Europe, Aged

## Abstract

**Background:**

There is growing evidence to suggest unemployment has a role in the development and incidence of cardiovascular disease. This study explores the contribution of breaks in employment to the development of hypertension, a key risk factor for coronary heart disease.

**Methods:**

We use data from the Survey of Health, Ageing, and Retirement in Europe to estimate the association between gaps in employment of 6 months or more (‘Not Working’, NW) and the incidence of hypertension in 9,985 individuals aged 50 or over across 13 European countries. Life history information including transitions in and out of employment was used to create a panel dataset where each visit represented one year of life between age 30 and incident hypertension or censoring (whichever came first). Pooled logistic models estimated the odds of hypertension according to the experience of not working, controlling for age at interview, age at each visit, gender, childhood socio-economic position, and country.

**Results:**

We consistently found no association between NW and hypertension, irrespective of the metrics used in defining the exposure or model specification.

**Conclusion:**

There is the possibility of bias contributing to the null findings. However, given the relatively consistent evidence for an association between unemployment and cardiovascular outcomes in the literature, our results suggest there may be mechanisms - outside of hypertension – that have a comparatively greater contribution to this association.

## Background

At present, more than 10% of the adult population of the European Union is unemployed, representing nearly 26 million people [1]. Even in countries with higher rates of employment, breaks between jobs are common; for example, most US adults experience at least one episode of unemployment by middle age [2]. Some researchers hypothesize that (among other mechanisms) these episodes are physiologically stressful, such that there may be somatic consequences to gaps in employment [3].

There is a large body of research exploring the connection between working life and health, including the examination of employment status and episodes of unemployment on health and mortality [4,5]. Many of these studies focus on cardiovascular disease (CVD), likely reflecting the high prevalence and mortality of its associated conditions. However, these investigations have been largely cross-sectional in design and are more vulnerable to biases, including reverse causation. Of the studies that ensure the temporal sequence of the exposure and outcome is maintained, the findings are fairly consistent. For example, the cohort study by Dupre et al. using data from the US Health and Retirement Study reported significant associations between unemployment status, job losses and episodes of not working with the incidence of acute cardiovascular events [6]. They found that those experiencing 4 or more job losses over the follow-up period had a 63% increase in the risk of acute myocardial infarction compared with those who had had none (HR 1.63 95% CI 1.29, 2.07). Similarly, Gallo and colleagues found that older workers who lost their jobs involuntarily had more than twice the risk of myocardial infarction and stroke than those who remained employed (HR 2.48 95% CI 1.49, 4.14) [5].

It is likely that there are differences between the US samples of these studies and European populations. Nonetheless, it is useful to consider the etiological theories for this association. Firstly, the stress of workplace factors and unemployment may directly affect the development of coronary heart disease mediators (such as atherosclerosis or metabolic syndrome) through dysregulation of neuroendocrine pathways [7,8]. There is still much to clarify about these biological mechanisms, and there is substantial ongoing research in this area. Secondly, lifestyle factors (such as alcohol intake, smoking, obesity) may vary with workplace variables and employment status, such that the impact of unemployment on CVD is mediated by these variables [8]. Thirdly, the effect of job loss on socioeconomic status may have independent negative health impacts [8]. In reality, it is likely that all of these mechanisms (and potentially other still unknown pathways) have a role.

Hypertension is a key mediator for myocardial infarction and other manifestations of ischemic heart disease, and a risk factor for (among others) the often-fatal conditions of stroke, peripheral vascular and kidney disease. Additionally, hypertension is highly prevalent worldwide, affecting around a third of those aged 30 years and over in European Union countries, and more than 200 million in greater Europe [9]. Lawes et al. determined that high blood pressure was responsible for the premature deaths of 7.6 million people worldwide in 2001, and that around 50% of all cases of stroke and ischemic heart disease were attributable to this factor [10]. Yet hypertension is both modifiable and potentially avoidable, identified as ‘the world’s most prevalent preventable condition’ by the World Health Organization [11]. Despite this – and despite the increasing body of evidence around employment and CVD - there is little published research considering how workforce factors such as unemployment may contribute to the etiology of this condition. There are cross-sectional studies that describe associations between unemployment and hypertension, [12,13] but findings from longitudinal investigations are mixed. Janlert et al. described increasing blood pressure according to the number of days unemployed in their study of Swedish workers, however this study involved 297 participants only [14]. Kasl et al. found short-term elevations in serum cholesterol following job loss, but no change in blood pressure [15].

This study extrapolated current theories around the contribution of unemployment to myocardial infarction to the outcome hypertension, exploring the hypothesis that breaks in employment may increase the incidence of hypertension. We used the Survey of Health, Ageing, and Retirement in Europe (SHARE), which contains information on physical and mental health (including both self-rated and objective measures) and social/economic functioning for population representative households containing at least one individual aged 50 or over. The third wave of this survey, termed SHARELIFE, also includes retrospective information such as employment history and workforce factors. We used three waves of this survey to create a panel dataset incorporating job and health information over the life-course.

## Methods

### Data source

We assembled data on non-institutionalized adults aged 50 years and older and their age-eligible spouses from SHARE. We used the first three waves of SHARE, which included nearly 34,000 participants from 14 countries. Participants from Austria, Belgium, Denmark, France, Germany, Greece, Italy, the Netherlands, Spain, Sweden, and Switzerland were first surveyed in 2004. Samples from the Czech Republic, Ireland, and Poland were incorporated in 2006-07. The third wave surveyed these participants again in 2008/9. Further details on sampling methodologies for SHARE are available in Borsch-Supan et al. [16-18].

SHARELIFE provides information on employment over participants’ lifetimes, including the timing of new jobs and whether there were breaks in employment. When this dataset was merged with that of wave 1 or 2 of SHARE, we were able to incorporate health data, including the diagnoses of clinical conditions and their timing. Using this information and after discretizing by time, a panel dataset was created with each year of life from the age of 30 corresponding to one visit. We focused on this time period to avoid potential bias from breaks in employment after finishing education. Subjects were censored at the age of interview if they were free of the outcome.

Individuals aged 50 years or over with a history of paid employment when aged 30 or above, who had participated in both waves 1 and 3 of the SHARE survey or both waves 2 and 3 were eligible for the study (n = 11,683). Individuals were excluded if they had history of employment in the armed forces (n = 346), were diagnosed with hypertension aged 29 or younger (n = 211), or had missing or implausible data in key variables (n = 1141). A sample of n = 9,985 remained for analysis.

### Measures

Participants were defined as ‘working’ or ‘not working’ (NW, the exposure) for each year between the age of 30 and censoring/outcome. Three sets of variables were used to create the NW indicator, and define the age at which the subject commenced each specific episode of NW. Firstly, the ‘year ended job’ measure (“in which year did you stop doing this job?”, question RE026_1-19). Secondly, participants could indicate a break in employment of 6 months or more between this job and the next with option 2 of question RE032_1-19; and finally, the participants’ year of birth. The corresponding visit, and each subsequent one, was defined as a year or part thereof not working (NW = 1). When the individual commenced their next paid job (as defined by “in which year did you start your next paid job?”, question RE011_1-20), the NW indicator became 0 and they were no longer classified as ‘not working’. Leave from work associated with the birth of child was classified separately in SHARELIFE. To be consistent with previous publications (e.g. 6), we applied no additional criteria to the reason for not working.

The exposure was evaluated in a number of ways, all of which were lagged by one visit to ensure episodes of NW were temporally prior to incidence of hypertension. We examined the cumulative count of the number of visits in which the participant was recorded as NW over the course of their follow-up - as a continuous variable, a 4 category term, and as being ‘ever NW’ (i.e. a NW count of 1 or more). Secondly, we used the proportion of follow-up time spent in a NW state. That is, at each visit, the cumulative number of visits with NW = 1 was divided by the number of years that subjects had been in the study.

The outcome ‘diagnosed hypertension’ was defined according to the SHARE question “Has a doctor ever told you that you had…. high blood pressure or hypertension” and “About how old were you when you were first told by a doctor that you had high blood pressure?”. These variables enabled the year in which a participant was diagnosed with hypertension to be identified, and the outcome was recorded at the corresponding visit in the panel dataset. Self-report data for hypertension has reasonable specificity (that is, few people report they have hypertension if in fact they do not), however like many other conditions, it may underestimate the true prevalence [19].

Information on covariates was obtained from the SHARELIFE questionnaire. These included age at interview (modeled as a continuous number of years), age at each visit (time varying continuous variable), gender, education (age at completion of full-time education, in years), and country indicator. Childhood socio-economic position (SEP) was measured using the occupation of the household breadwinner during the participants’ childhood. This was categorized according to International Standard Classification of Occupations-88 groups (1 -9), [20] and used as a 4 category variable (with 1 representing higher socio-economic position – used as the reference category - and 4 the lowest).

### Statistical analysis

Unadjusted means/proportions were used to compare characteristics of the sample (age, gender, education, childhood SEP, distribution of exposure) according to gender and country. Pooled logistic models compared the odds of hypertension according to the exposure NW. That is, at each visit for each individual, the odds of hypertension with respect to employment state were modeled, and these multiple estimates then combined into one pooled estimate [21]. To control for potential confounding, the adjusted model included terms for age at time of interview*,* age at visit*,* gender*,* childhood SEP, and country*.* The STATA code used for these analyses is available in Additional file 1.

## Results

Table 1 gives the distribution of covariates and the exposure NW according to gender for the sample of 9,985 participants. Overall, there were fewer men in the study than women (46% compared with 54%). There were no substantial differences between men and women with respect to age at interview, education, follow up duration, or childhood SEP. The distribution of the frequency of episodes of NW varied significantly between men and women, with 80% of men working consistently (0 NW episodes) compared with only 39% of women. Five or more episodes of NW were reported by 5% of men compared with 36% of women. Overall, women spent 15% of their follow up time not working whereas men spent only 3%. Further descriptive analyses (distribution of covariates according to country) are available in Additional file 2.

**Table 1:** Distribution of covariates and exposure according to gender

	**Male**	**Female**	**Total**
**N (%)**	4592 (46.0)	5393 (54.0)	9985
**Age (SD)**	66.1 (9.3)	64.8 (9.6)	65.4 (9.5)
**Education (SD)**	18.1 (5.7)	17.4 (5.4)	17.7 (5.6)
**Mean follow up, yrs (SD)**	30.8 (10.7)	29.2 (10.7)	30.0 (10.7)
**Childhood SES (%)**^**a**^	500 (10.9)	564 (10.5)	1064 (10.7)
**1**			
**2**	248 (5.4)	280 (5.2)	528 (5.3)
**3**	3040 (66.2)	3623 (67.2)	6663 (66.7)
**4**	804 (17.5)	926 (17.2)	1730 (17.3)
**Country (%) ** **Austria**	90 (2.0)	142 (2.6)	232 (2.3)
**Belgium**	433 (9.4)	421 (7.8)	854 (8.6)
**Czech Republic**	315 (6.9)	469 (8.7)	784 (7.9)
**Denmark**	569 (12.4)	719 (13.3)	1288 (12.9)
**France**	456 (9.9)	568 (10.5)	1024 (10.3)
**Germany**	350 (7.6)	449 (8.3)	799 (8.0)
**Greece**	266 (5.8)	283 (5.3)	549 (5.5)
**Italy**	389 (8.5)	282 (5.2)	671 (6.7)
**Netherlands**	379 (8.3)	502 (9.3)	881 (8.8)
**Poland**	290 (6.3)	379 (7.0)	669 (6.7)
**Spain**	292 (6.4)	239 (4.4)	531 (5.3)
**Switzerland**	453 (9.9)	591 (11.0)	1044 (10.5)
**Sweden**	310 (6.8)	349 (6.5)	659 (6.6)
**Count of episodes**^**b**^	N = 4573	N = 5371	N = 9944
**0**	3683 (80.5)	2080 (38.7)	5763 (58.0)
**1**	289 (6.3)	436 (8.1)	725 (7.3)
**2**	178 (3.9)	356 (6.6)	534 (5.4)
**3**	112 (2.5)	314 (5.9)	426 (4.3)
**4**	77 (1.7)	274 (5.1)	351 (3.5)
**5 or more**	234 (5.1)	1911 (35.6)	2145 (21.6)
**Mean number of episodes (SD, max)**	0.8 (2.4, 31)	4.5 (5.9, 36)	2.8 (5.0, 36)
**Proportion of time not working, mean (SD, max)**	0.03 (0.08, 0.96)	0.15 (0.21, 0.97)	0.1 (0.17, 0.97)

^a^Category 1 = ‘legislators, senior officials and managers’ and ‘professionals’ (ISCO-88 major groups 1 and 2); category 2=’technicians and associate professionals’ (ISCO-88 major group 3); category 3 = ‘clerks’, ‘service, shop or market sales worker’, ‘skilled agricultural or fishery worker’, ‘craft or related trades workers’, ‘plant/machine operator or assembler’ (ISCO-88 major groups 4, 5, 6, 7 and 8); and category 4=’elementary occupation’ (ISCO-88 major group 9). ^b^This total is less as 41 people diagnosed with hypertension at age 30 (the start of follow up). Given exposure is lagged by one visit, these subjects are not considered in this table.

The multivariable results showed that there was no meaningful impact of NW on the incidence of hypertension, with the odds ratios for all adjusted models clustered around 1.00 (given in Table 2). When NW was explored as a continuous variable, the increased odds of hypertension associated with a unit change in NW were almost perfectly null: 1.005 (95% CI 0.997 - 1.014). The pattern of results was not sensitive to the form of the NW exposure. That is, null findings were demonstrated when NW was modeled as a categorical or continuous count variable (even with a quadratic term included) of NW episodes, as the cumulative ‘proportion of time in NW state’, and as a dichotomous ‘ever NW’ variable. The null finding was also robust to the specification of the model (with respect to the inclusion/exclusion of the covariates listed), and sex-stratified models were similarly consistent with no adjusted association between NW and diagnosis of hypertension.

**Table 2:** Odds ratio of hypertension according to not working, adjusted model

	**Male**	**Female**	**Total**^**a**^
**Cumulative number of NW episodes, continuous**	1.00 (0.97 – 1.02)	1.00 (0.99 – 1.01)	1.01 (1.00 - 1.01)
**Cumulative number of NW episodes, 4 strata**			
**0**	Reference		
**1 and 2**	1.08 (0.90 – 1.31)	0.99 (0.86 -1.15)	1.04 (0.92 - 1.16)
**3 and 4**	1.21 (0.91 – 1.61)	0.97 (0.82 – 1.14)	1.03 (0.90 – 1.19)
**5+**	0.96 (0.73 – 1.27)	1.04 (0.93 – 1.18)	1.07 (0.97 – 1.19)
**Proportion of time in NW state**	0.72 (0.42 – 1.24)	0.99 (0.81 – 1.21)	0.98 (0.82 – 1.18)
**Ever NW compared with continuously employed**	1.08 (0.93 – 1.24)	1.01 (0.92 – 1.12)	1.05 (0.97 – 1.14)

Adjusted model: age at visit (continuous variable), age at interview (continuous), age finished education (continuous), childhood socio-economic position (4 categories), country (13 dummies). NW= Not Working.

^a^as per Model 1, with addition of gender term. Given the exposure is lagged by one visit, these analyses do not include the data from 41 people diagnosed with hypertension at age 30 (the start of follow up).

## Discussion

Our study uses data from 9,985 individuals aged 50 and over from 13 European countries to explore the association between episodes of NW and the development of hypertension. Irrespective of model specification and the metric of the exposure, our study finds no evidence that gaps between jobs of 6 months or more during adult life increase the odds of hypertension diagnosis.

This study adds to a growing body of research focused on the development and morbidity of cardiovascular disease. However, it has some limitations. Firstly, the exposure NW does not discriminate between those unwillingly unemployed and job seeking and those who may be out of the workforce for other reasons (such as incarceration). Secondly, the outcome hypertension is assessed retrospectively based on self-reported doctors diagnosis and may be mismeasured, although there is evidence to support the validity of survey data for this condition [19]. However, this issue is likely to be of lesser significance compared to the proportion of hypertension in this population that is undiagnosed. That is, there is misclassification of participants as ‘not hypertensive’ when in fact they are – if this bias was equally likely to affect those NW as working, it would underestimate any association between NW and the outcome. However, it is probable that access to medical care (and so likelihood of diagnosis) is related to employment status. In which case, this bias could act to either over or under-estimate the odds ratio. It is also possible that an association between NW and blood pressure exists, but that any elevations in systolic/diastolic measurements do not meet the criteria used to diagnose hypertension. That is, the definition of our outcome is too blunt to detect an association should one exist. Third, there may be confounding from other variables not included in our models: for example, health behaviors associated with unemployment (such as alcohol intake or diet) may have a role. However this is unlikely as unhealthy lifestyle factors would induce an association between NW and hypertension, as opposed to bias it towards the null. Also, we found very little impact of confounding in our analyses. Fourthly, like all surveys, nonresponse bias is possible. Participation rates across the countries were variable, with the proportion of respondents as low as 39% in Switzerland. Nonetheless, the overall response rate in wave 1 was 62%, [16] a reasonable proportion that is comparable to those from other European surveys (such as the European Social Survey).

## Conclusions

The relationship between workforce factors and health is complex. There are multiple pathways acting across time, and variables with roles as both confounders and mediators. Any association is also highly context-specific, with cultural and political variables likely to have important contributions. This study adds to current work by exploring a relatively under-researched issue, the association between unemployment and hypertension. We created a discretized panel dataset that tracked job transitions and incident hypertension in 9,985 individuals across 13 countries and employing pooled logistic models to control for baseline and time-varying covariates. Overall, we found no evidence of an impact of NW on the diagnosis of hypertension.

There are two broad explanations for our findings. The first is that there is truly no association between episodes of NW and the development of hypertension in our sample of older European workers. Given the relatively consistent evidence for an association between unemployment and cardiovascular outcomes, this suggests that there are other mechanisms - outside of hypertension – that are of greater importance in this relationship. Longitudinal studies with robust mediation analyses may help to identify the key component causes. Effect modification may also have a role. Our sample was made up of those aged 50 or over at the time of the interview, however some studies have found the associations between work stress and coronary heart disease to be stronger in younger cohorts [8]. It is also possible that in our sample, breaks between jobs were not physiologically damaging. That is, the findings of those researchers demonstrating adverse cardiovascular outcomes in association with unemployment are not generalizable to our group of older European workers. A second explanation to our findings is that there is an association in our sample, but various factors (such as misclassification of the outcome hypertension) may have biased the findings towards the null.

This study contributes to the growing research base around the interplay between employment and health. However, there are several pathways for future research. For example, there is an indication for methodologically-focused studies to explore the role of mediators and time-dependent confounders, and the extent of their impact. Additionally, prospective studies may be useful to obtain more precise information on the timing and impact of gradually progressive conditions, such as increases in blood pressure or weight, with respect to transitions in employment.

## Competing interests

The authors declare that they have no competing interest.

## Authors’ contributions

JRS, AN, and JK all contributed to the conception and design of the study. JRS and AN had full access to all of the data in the study; JRS takes responsibility for the integrity of the data and the accuracy of the data analysis. All authors (JRS, AN, and JK) contributed to the interpretation of results. JRS drafted the article and all authors reviewed and edited the article. All authors approve of the final version of the manuscript and have taken due care to ensure the integrity of this work.

## Pre-publication history

The pre-publication history for this paper can be accessed here:

http://www.biomedcentral.com/1471-2458/14/536/prepub

## Supplementary Material

Additional file 1 Creation of exposure and outcome variables – STATA code. Click here for file

Additional file 2 **Distribution of key covariates according to country (supplementary material).** a Category 1 = ‘legislators, senior officials and managers’ and ‘professionals’ (ISCO-88 major groups 1 and 2); category 2 = ’technicians and associate professionals’ (ISCO-88 major group 3); category 3 = ‘clerks’, ‘service, shop or market sales worker’, ‘skilled agricultural or fishery worker’, ‘craft or related trades workers’, ‘plant/machine operator or assembler’ (ISCO-88 major groups 4, 5, 6, 7 and 8); and category 4 = ’elementary occupation’ (ISCO-88 major group 9). Data obtained from the Survey of Health, Ageing, and Retirement in Europe. Click here for file
