# Supplementary material for: Working and hypertension: gaps in employment not associated with increased risk in 13 European countries, a retrospective cohort study
Source: BMC Public Health. 2014 May 30;14:536. doi: 10.1186/1471-2458-14-536 (PMC4055277; doi:10.1186/1471-2458-14-536)
Supplement: Additional file 1 — Creation of exposure and outcome variables – STATA code. [file 1471-2458-14-536-S1.docx]

**STATA CODE**

Note: This file refers to data created from merged SHARELIFE and SHARE (waves 1 and 2) datasets.

**A. CREATION OF EXPOSURE AND OUTCOME VARIABLES**

‘w1’ denotes variable from wave 1 of SHARE; ‘w2’ refers to the equivalent wave 2 variable; ‘sl’ refers to a SHARELIFE variable. ‘Agev2’ = the age at interview, age_fin_edu = age finished education, occbw_cat = childhood socioeconomic position, country=country, age0=age at visit, mal=male gender.

//1. Gap in employment variable

generate gap=1 if sl_re032_1==2 | sl_re032_2==2

replace gap=1 if sl_re032_3==2 | sl_re032_4==2 |sl_re032_5==2 | sl_re032_6==2

replace gap=1 if sl_re032_7==2 | sl_re032_8==2 |sl_re032_9==2 | sl_re032_10==2

replace gap=1 if sl_re032_11==2 | sl_re032_12==2 |sl_re032_13==2 | sl_re032_14==2

replace gap=1 if sl_re032_15==2 | sl_re032_16==2 |sl_re032_17==2 | sl_re032_18==2

replace gap=1 if sl_re032_19==2

replace gap=0 if gap==.

//2. Hypertension variable (HT), the outcome of interest

generate HT=1 if w2ph006d2==1 | w1ph006d2==1

replace HT=0 if HT==.

//3. Age at diagnosis

generate ageHT=w1ph009_2

replace ageHT=w2ph009_2 if w1ph009_2==.

//4. Age at each gap in employment

//generate age of end of job (i.e. the job before the gap of > 6 months)

//make job ineligible if age<14

gen gap1=.

replace gap1=(sl_re026_1 - yearofbirth) if sl_re026_1~=.

replace gap1=999 if gap1<14

gen agegap1=.

replace agegap1 = gap1 if (sl_re032_1==2 & sl_re026_1~=9997) & gap1~=999

gen gap2=.

replace gap2=(sl_re026_2 - yearofbirth) if sl_re026_2~=.

replace gap2=999 if gap2<14

gen agegap2=.

replace agegap2 = gap2 if (sl_re032_2==2 & sl_re026_2~=9997) & gap2~=999

replace agegap1=agegap2 if gap1==999

gen gap3=.

replace gap3=(sl_re026_3 - yearofbirth) if sl_re026_3~=.

replace gap3=999 if gap3<14

gen agegap3=.

replace agegap3 = gap3 if (sl_re032_3==2 & sl_re026_3~=9997) & gap3~=999

replace agegap2=agegap3 if gap2==999

gen gap4=.

replace gap4=(sl_re026_4 - yearofbirth) if sl_re026_4~=.

replace gap4=999 if gap4<14

gen agegap4=.

replace agegap4 = gap4 if (sl_re032_4==2 & sl_re026_4~=9997) & gap4~=999

replace agegap3=agegap4 if gap3==999

gen gap5=.

replace gap5=(sl_re026_5 - yearofbirth) if sl_re026_5~=.

replace gap5=999 if gap5<14

gen agegap5=.

replace agegap5 = gap5 if (sl_re032_5==2 & sl_re026_5~=9997) & gap5~=999

replace agegap4=agegap5 if gap4==999

gen gap6=.

replace gap6=(sl_re026_6 - yearofbirth) if sl_re026_6~=.

replace gap6=999 if gap6<14

gen agegap6=.

replace agegap6 = gap6 if (sl_re032_6==2 & sl_re026_6~=9997) & gap6~=999

replace agegap5=agegap6 if gap5==999

gen gap7=.

replace gap7=(sl_re026_7 - yearofbirth) if sl_re026_7~=.

replace gap7=999 if gap7<14

gen agegap7=.

replace agegap7 = gap7 if (sl_re032_7==2 & sl_re026_7~=9997) & gap7~=999

replace agegap6=agegap7 if gap6==999

gen gap8=.

replace gap8=(sl_re026_8 - yearofbirth) if sl_re026_8~=.

replace gap8=999 if gap8<14

gen agegap8=.

replace agegap8 = gap8 if (sl_re032_8==2 & sl_re026_8~=9997) & gap8~=999

replace agegap7=agegap8 if gap7==999

gen gap9=.

replace gap9=(sl_re026_9 - yearofbirth) if sl_re026_9~=.

replace gap9=999 if gap9<14

gen agegap9=.

replace agegap9 = gap9 if (sl_re032_9==2 & sl_re026_9~=9997) & gap9~=999

replace agegap8=agegap9 if gap8==999

gen gap10=.

replace gap10=(sl_re026_10 - yearofbirth) if sl_re026_10~=.

replace gap10=999 if gap10<14

gen agegap10=.

replace agegap10 = gap10 if (sl_re032_10==2 & sl_re026_10~=9997) & gap10~=999

replace agegap9=agegap10 if gap9==999

gen gap11=.

replace gap11=(sl_re026_11 - yearofbirth) if sl_re026_11~=.

replace gap11=999 if gap11<14

gen agegap11=.

replace agegap11 = gap11 if (sl_re032_11==2 & sl_re026_11~=9997) & gap11~=999

replace agegap10=agegap11 if gap10==999

gen gap12=.

replace gap12=(sl_re026_12 - yearofbirth) if sl_re026_12~=.

replace gap12=999 if gap12<14

gen agegap12=.

replace agegap12 = gap12 if (sl_re032_12==2 & sl_re026_12~=9997) & gap12~=999

replace agegap11=agegap12 if gap11==999

gen gap13=.

replace gap13=(sl_re026_13 - yearofbirth) if sl_re026_13~=.

replace gap13=999 if gap13<14

gen agegap13=.

replace agegap13 = gap13 if (sl_re032_13==2 & sl_re026_13~=9997) & gap13~=999

replace agegap12=agegap13 if gap12==999

gen gap14=.

replace gap14=(sl_re026_14 - yearofbirth) if sl_re026_14~=.

replace gap14=999 if gap14<14

gen agegap14=.

replace agegap14 = gap14 if (sl_re032_14==2 & sl_re026_14~=9997) & gap14~=999

replace agegap13=agegap14 if gap13==999

gen gap15=.

replace gap15=(sl_re026_15 - yearofbirth) if sl_re026_15~=.

replace gap15=999 if gap15<14

gen agegap15=.

replace agegap15 = gap15 if (sl_re032_15==2 & sl_re026_15~=9997) & gap15~=999

replace agegap14=agegap15 if gap14==999

gen gap16=.

replace gap16=(sl_re026_16 - yearofbirth) if sl_re026_16~=.

replace gap16=999 if gap16<14

gen agegap16=.

replace agegap16 = gap16 if (sl_re032_16==2 & sl_re026_16~=9997) & gap16~=999

replace agegap15=agegap16 if gap15==999

gen gap17=.

replace gap17=(sl_re026_17 - yearofbirth) if sl_re026_17~=.

replace gap17=999 if gap17<14

gen agegap17=.

replace agegap17 = gap17 if (sl_re032_17==2 & sl_re026_17~=9997) & gap17~=999

replace agegap16=agegap17 if gap16==999

gen gap18=.

replace gap18=(sl_re026_18 - yearofbirth) if sl_re026_18~=.

replace gap18=999 if gap18<14

gen agegap18=.

replace agegap18 = gap18 if (sl_re032_18==2 & sl_re026_18~=9997) & gap18~=999

replace agegap17=agegap18 if gap17==999

gen gap19=.

replace gap19=(sl_re026_19 - yearofbirth) if sl_re026_19~=.

replace gap19=999 if gap19<14

gen agegap19=.

replace agegap19 = gap19 if (sl_re032_19==2 & sl_re026_19~=9997) & gap19~=999

replace agegap18=agegap19 if gap18==999

//5. Age at start of each job 1- 20

generate agejob1=.

generate a1=.

replace a1=(sl_re011_1 - yearofbirth) if sl_re011_1~=.

replace a1=999 if a1<14

replace agejob1=a1 if a1~=999

generate agejob2=.

generate a2=.

replace a2=(sl_re011_2 - yearofbirth) if sl_re011_2~=.

replace a2=999 if a2<14

replace agejob2=a2 if a2~=999

replace agejob1=agejob2 if a1==999

generate agejob3=.

generate a3=.

replace a3=(sl_re011_3 - yearofbirth) if sl_re011_3~=.

replace a3=999 if a3<14

replace agejob3=a3 if a3~=999

replace agejob2=agejob3 if a2==999

generate agejob4=.

gen a4=.

replace a4=(sl_re011_4 - yearofbirth) if sl_re011_4~=.

replace a4=999 if a4<14

replace agejob4=a4 if a4~=999

replace agejob3=agejob4 if a3==999

generate agejob5=.

gen a5=.

replace a5=(sl_re011_5 - yearofbirth) if sl_re011_5~=.

replace a5=999 if a5<14

replace agejob5=a5 if a5~=999

replace agejob4=agejob5 if a4==999

generate agejob6=.

generate a6=.

replace a6=(sl_re011_6 - yearofbirth) if sl_re011_6~=.

replace a6=999 if a6<14

replace agejob6=a6 if a6~=999

replace agejob5=agejob6 if a5==999

generate agejob7=.

generate a7=.

replace a7=(sl_re011_7 - yearofbirth) if sl_re011_7~=.

replace a7=999 if a7<14

replace agejob7=a7 if a7~=999

replace agejob6=agejob7 if a6==999

generate agejob8=.

generate a8=.

replace a8=(sl_re011_8 - yearofbirth) if sl_re011_8~=.

replace a8=999 if a8<14

replace agejob8=a8 if a8~=999

replace agejob7=agejob8 if a7==999

generate agejob9=.

generate a9=.

replace a9=(sl_re011_9 - yearofbirth) if sl_re011_9~=.

replace a9=999 if a9<14

replace agejob9=a9 if a9~=999

replace agejob8=agejob9 if a8==999

generate agejob10=.

generate a10=.

replace a10=(sl_re011_10 - yearofbirth) if sl_re011_10~=.

replace a10=999 if a10<14

replace agejob10=a10 if a10~=999

replace agejob9=agejob10 if a9==999

generate agejob11=.

generate a11=.

replace a11=(sl_re011_11 - yearofbirth) if sl_re011_11~=.

replace a11=999 if a11<14

replace agejob11=a11 if a11~=999

replace agejob10=agejob11 if a10==999

generate agejob12=.

generate a12=.

replace a12=(sl_re011_12 - yearofbirth) if sl_re011_12~=.

replace a12=999 if a12<14

replace agejob12=a12 if a12~=999

replace agejob11=agejob12 if a11==999

generate agejob13=.

generate a13=.

replace a13=(sl_re011_13 - yearofbirth) if sl_re011_13~=.

replace a13=999 if a13<14

replace agejob13=a13 if a13~=999

replace agejob12=agejob13 if a12==999

generate agejob14=.

generate a14=.

replace a14=(sl_re011_14 - yearofbirth) if sl_re011_14~=.

replace a14=999 if a14<14

replace agejob14=a14 if a14~=999

replace agejob13=agejob14 if a13==999

generate agejob15=.

generate a15=.

replace a15=(sl_re011_15 - yearofbirth) if sl_re011_15~=.

replace a15=999 if a15<14

replace agejob15=a15 if a15~=999

replace agejob14=agejob15 if a14==999

generate agejob16=.

generate a16=.

replace a16=(sl_re011_16 - yearofbirth) if sl_re011_16~=.

replace a16=999 if a16<14

replace agejob16=a16 if a16~=999

replace agejob15=agejob16 if a15==999

generate agejob17=.

generate a17=.

replace a17=(sl_re011_17 - yearofbirth) if sl_re011_17~=.

replace a17=999 if a17<14

replace agejob17=a17 if a17~=999

replace agejob16=agejob17 if a16==999

generate agejob18=.

generate a18=.

replace a18=(sl_re011_18 - yearofbirth) if sl_re011_18~=.

replace a18=999 if a18<14

replace agejob18=a18 if a18~=999

replace agejob17=agejob18 if a17==999

generate agejob19=.

gen a19=.

replace a19=(sl_re011_19 - yearofbirth) if sl_re011_19~=.

replace a19=999 if a19<14

replace agejob19=a19 if a19~=999

replace agejob18=agejob19 if a18==999

drop a1 a2 a3 a4 a5 a6 a7 a8 a9 a10 a11 a12 a13 a14 a15 a16 a17 a18 a19

drop gap1 gap2 gap3 gap4 gap5 gap6 gap7 gap8 gap9 gap10 gap11 gap12 gap13 gap14 gap15 gap16 gap17 gap18 gap19

//5. Censoring

//censor at the age of interview

generate agecensor=.

replace agecensor=age if (agev2-ageHT~=0)

//6.End of follow up: determined by either outcome or censoring (interview)

generate age_out=.

replace age_out=(ageHT + 1) if (ageHT~=. & ageHT<agecensor)

replace age_out=(agecensor + 1) if agecensor<ageHT

//7. Start of follow-up

generate age_in=1

**B. CREATE DISCRETIZED DATASET**

stset duration, failure(HT) id(id)

stsplit year, every(1)

1.//Create ‘age at visit’ variable (age0)

generate age0=year + age_in

2.//Censor variable

generate censor=.

replace censor=1 if agecensor==age0

replace censor=0 if censor==.

3.//Unemployed variable per visit, ‘UE’

generate UE=.

replace UE=1 if agegap1==age0

replace UE=1 if agegap2==age0

replace UE=1 if agegap3==age0

replace UE=1 if agegap4==age0

replace UE=1 if agegap5==age0

replace UE=1 if agegap6==age0

replace UE=1 if agegap7==age0

replace UE=1 if agegap8==age0

replace UE=1 if agegap9==age0

replace UE=1 if agegap10==age0

replace UE=1 if agegap11==age0

replace UE=1 if agegap12==age0

replace UE=1 if agegap13==age0

replace UE=1 if agegap14==age0

replace UE=1 if agegap15==age0

replace UE=1 if agegap16==age0

replace UE=1 if agegap17==age0

replace UE=1 if agegap18==age0

replace UE=1 if agegap19==age0

replace UE=0 if agejob1==age0

replace UE=0 if agejob2==age0

replace UE=0 if agejob3==age0

replace UE=0 if agejob4==age0

replace UE=0 if agejob5==age0

replace UE=0 if agejob6==age0

replace UE=0 if agejob7==age0

replace UE=0 if agejob8==age0

replace UE=0 if agejob9==age0

replace UE=0 if agejob10==age0

replace UE=0 if agejob11==age0

replace UE=0 if agejob12==age0

replace UE=0 if agejob13==age0

replace UE=0 if agejob14==age0

replace UE=0 if agejob15==age0

replace UE=0 if agejob16==age0

replace UE=0 if agejob17==age0

replace UE=0 if agejob18==age0

replace UE=0 if agejob19==age0

stfill UE, forward

4.//lagged unemployment variable by one visit

generate UELAG=.

replace UELAG=1 if agegap1==age0-1

replace UELAG=1 if agegap2==age0-1

replace UELAG=1 if agegap3==age0-1

replace UELAG=1 if agegap4==age0-1

replace UELAG=1 if agegap5==age0-1

replace UELAG=1 if agegap6==age0-1

replace UELAG=1 if agegap7==age0-1

replace UELAG=1 if agegap8==age0-1

replace UELAG=1 if agegap9==age0-1

replace UELAG=1 if agegap10==age0-1

replace UELAG=1 if agegap11==age0-1

replace UELAG=1 if agegap12==age0-1

replace UELAG=1 if agegap13==age0-1

replace UELAG=1 if agegap14==age0-1

replace UELAG=1 if agegap15==age0-1

replace UELAG=1 if agegap16==age0-1

replace UELAG=1 if agegap17==age0-1

replace UELAG=1 if agegap18==age0-1

replace UELAG=1 if agegap19==age0-1

replace UELAG=0 if agejob1==age0-1

replace UELAG=0 if agejob2==age0-1

replace UELAG=0 if agejob3==age0-1

replace UELAG=0 if agejob4==age0-1

replace UELAG=0 if agejob5==age0-1

replace UELAG=0 if agejob6==age0-1

replace UELAG=0 if agejob7==age0-1

replace UELAG=0 if agejob8==age0-1

replace UELAG=0 if agejob9==age0-1

replace UELAG=0 if agejob10==age0-1

replace UELAG=0 if agejob11==age0-1

replace UELAG=0 if agejob12==age0-1

replace UELAG=0 if agejob13==age0-1

replace UELAG=0 if agejob14==age0-1

replace UELAG=0 if agejob15==age0-1

replace UELAG=0 if agejob16==age0-1

replace UELAG=0 if agejob17==age0-1

replace UELAG=0 if agejob18==age0-1

replace UELAG=0 if agejob19==age0-1

stfill UELAG, forward

5.//start follow up from age 30

sort id year

drop if age0<30

6.//Make those not working at start of follow up unemployed (i.e. if before their first job)

replace UE=1 if (age0<agejob1 & UE==.)

replace UELAG=1 if (agejob1<(age0+1) & UELAG==.)

replace UELAG=1 if age0<agejob1 & UELAG==.

7.//lose info of first visit as lagged variable

replace UELAG=. if age0==30

8.//make HT a 0/1 variable i.e. give it a value for each visit, currently only positive at age HT diagnosed

replace HT=0 if HT==.

9.//UE into different definitions of Not Working

//A. Count of episodes of UE, lagged by 1 visit

gen cumUE_l=.

by id: replace cumUE_l=sum(UELAG)

replace cumUE_l=. if age0==30

//B. Strata of Not Working

gen strataUE=.

replace strataUE=0 if cumUE_l==0

replace strataUE=1 if cumUE_l==1 | cumUE_l==2

replace strataUE=2 if cumUE_l==3 | cumUE_l==4

replace strataUE=3 if cumUE_l>4

replace strataUE=. if cumUE_l==.

//C. Proportion of time (follow-up duration) spent Not Working

gen UE_prop =.

by id: replace UE_prop=(cumUE_l)/(age0-29) if cumUE_l~=0

replace UE_prop=0 if UE_prop==.

replace UE_prop=. if age0==30

//D. Ever ‘Not Working’ over follow-up

gen UE_ever=.

replace UE_ever=1 if cumUE_l>=1

replace UE_ever=0 if cumUE_l<1

replace UE_ever=. if age0==30

**c. ANALYSIS**

//DISCRETE LOGISTIC LONGFORM

//Covariates: age at visit (Time Varying Confounder), age at interview (Time Fixed), mal (male gender), age finished education (TF), childhood SEP (TF), country (TF)

logistic HT i.strataUE age0 agev2 mal age_fin_edu i.occbw_cat i.country, cluster(id)

logistic HT cumUE_l age0 agev2 mal age_fin_edu i.occbw_cat i.country, cluster(id)

logistic HT UE_prop age0 agev2 mal age_fin_edu i.occbw_cat i.country, cluster(id)

logistic HT UE_ever age0 agev2 mal age_fin_edu i.occbw_cat i.country, cluster(id)
