# Supplementary material for: Working and hypertension: gaps in employment not associated with increased risk in 13 European countries, a retrospective cohort study
Source: BMC Public Health. 2014 May 30;14:536. doi: 10.1186/1471-2458-14-536 (PMC4055277; doi:10.1186/1471-2458-14-536)
Supplement: Additional file 2 — Distribution of key covariates according to country (supplementary material). a Category 1 = ‘legislators, senior officials and managers’ and ‘professionals’ (ISCO-88 major groups 1 and 2); category 2 = ’technicians and associate professionals’ (ISCO-88 major group 3); category 3 = ‘clerks’, ‘service, shop or market sales worker’, ‘skilled agricultural or fishery worker’, ‘craft or related trades workers’, ‘plant/machine operator or assembler’ (ISCO-88 major groups 4, 5, 6, 7 and 8); and category 4 = ’elementary occupation’ (ISCO-88 major group 9). Data obtained from the Survey of Health, Ageing, and Retirement in Europe. [file 1471-2458-14-536-S2.docx]

**APPENDIX 1: DISTRIBUTION OF KEY COVARIATES ACCORDING TO COUNTRY**

|  | **Austria** | **Belgium** | **Czech Republic** | **Denmark** | **France** | **Germany** | **Greece** | **Italy** | **Netherland** | **Poland** | **Spain** | | **Sweden** | **Switzerland** | | **Total** |
| --- | --- | --- | --- | --- | --- | --- | --- | --- | --- | --- | --- | --- | --- | --- | --- | --- |
| **N** | 232 | 854 | 784 | 1288 | 1024 | 799 | 549 | 671 | 881 | 669 | 531 | 1044 | | | 659 | 9985 |
| **Mean age at interview (SD)** | 68.4 (9.4) | 65.8 (10.2) | 64.5 (9.0) | 64.8  (9.8) | 66.1 (9.8) | 65.0  (8.9) | 64.3 (9.2) | 66.8 (9.2) | 64.6  (8.7) | 62.4 (8.9) | 65.9 (10.0) | 68.0  (9.2) | | | 64.6  (9.3) | 65.4 (9.5) |
| **% Male** | 38.8 | 50.7 | 40.2 | 44.2 | 44.5 | 43.8 | 48.5 | 58.0 | 43.0 | 43.3 | 55.0 | 43.4 | | | 47.0 | 46.0 |
| **Mean age finishing education (SD)** | 16.3 (4.2) | 17.7 (4.0) | 18.0  (2.8) | 19.5  (5.1) | 17.4 (4.6) | 18.9  (4.5) | 15.8 (5.9) | 14.5 (6.0) | 17.8  (4.6) | 16.8 (3.6) | 12.6 (6.0) | 20.1  (8.6) | | | 19.2  (3.9) | 17.7 (5.6) |
| **Childhood SES (%)** a  **1** | 19  (8.2) | 101 (11.8) | 46  (5.9) | 181  (14.1) | 138 (13.5) | 67  (8.4) | 28  (5.1) | 26 (3.9) | 141  (16.0) | 32  (4.8) | 23  (4.3) | 184 (17.6) | | | 78  (11.8) | 1064 (10.7) |
| **2** | 7  (3.0) | 52  (6.1) | 73  (9.3) | 55  (4.3) | 68  (6.6) | 49  (6.1) | 5  (0.9) | 17  (2.5) | 61  (6.9) | 19  (2.8) | 22  (4.1) | 58  (5.6) | | | 42  (6.4) | 528  (5.3) |
| **3** | 167 (72.0) | 403 (47.2) | 593 (75.6) | 791  (61.4) | 631 (61.6) | 610  (76.4) | 427 (77.8) | 399 (59.5) | 589  (66.9) | 551 (82.4) | 316 (59.5) | 706  (67.6) | | | 480  (72.8) | 6663  (66.7) |
| **4** | 39 (16.8) | 298 (34.9) | 72  (9.2) | 261  (20.3) | 187 (18.3) | 73  (9.1) | 89 (16.2) | 229 (34.1) | 90  (10.2) | 67 (10.0) | 170 (32.0) | 96  (9.2) | | | 59  (9.0) | 1730  (17.3) |
| **Mean follow-up, yrs (SD)** | 30.9  (12.0) | 30.3 (11.3) | 27.9  (9.9) | 30.1  (10.5) | 30.8 (11.1) | 28.4  (10.5) | 29.0 (9.7) | 30.3 (10.8) | 30.5  (10.0) | 26.3 (10.5) | 31.0 (11.2) | 32.5 (11.0) | | | 30.5  (10.2) | 30.0 (10.7) |

a Category 1= ‘legislators, senior officials and managers’ and ‘professionals’ (ISCO-88 major groups 1 and 2); category 2=’technicians and associate professionals’ (ISCO-88 major group 3); category 3= ‘clerks’, ‘service, shop or market sales worker’, ‘skilled agricultural or fishery worker’, ‘craft or related trades workers’, ‘plant/machine operator or assembler’ (ISCO-88 major groups 4, 5, 6, 7 and 8); and category 4=’elementary occupation’ (ISCO-88 major group 9).
